# Insecticidal and growth inhibitory activity of gut microbes isolated from adults of *Spodoptera litura* (Fab.)

**DOI:** 10.1186/s12866-022-02476-3

**Published:** 2022-03-10

**Authors:** Sarita Devi, Harvinder Singh Saini, Sanehdeep Kaur

**Affiliations:** 1 Department of Zoology, Guru Nanak Dev University, 143005 Amritsar, Punjab India; 2 Department of Microbiology, Guru Nanak Dev University, 143005 Amritsar, Punjab India

**Keywords:** *Spodoptera litura*, *Klebsiella pneumoniae* and *Pseudomonas paralactis*, Insecticidal potential, Microbial control

## Abstract

**Background:**

*Spodoptera litura* (Fab.) (Lepidoptera: Noctuidae) commonly known as tobacco caterpillar is a polyphagous pest that causes significant damage to many agricultural crops. The extensive use of chemical insecticides against *S. litura* has resulted in development of resistance. In order to find potential biocontrol agents, gut microbes were investigated for insecticidal potential. These microbes live in a diverse relationship with insects that may vary from beneficial to pathogenic.

**Results:**

*Enterococcus casseliflavus*, *Enterococcus mundtii*, *Serratia marcescens*, *Klebsiella pneumoniae*, *Pseudomonas paralactis* and *Pantoea brenneri* were isolated from adults of *S. litura.* Screening of these microbial isolates for insecticidal potential against *S. litura* showed higher larval mortality due to *K. pneumoniae* and *P. paralactis*. These bacteria also negatively affected the development of insect along with significant decline in relative growth and consumption rate as well as efficiency of conversion of ingested and digested food of insect. The bacteria significantly decreased the reproductive potential of insect. Perturbations in the composition of gut microbiome and damage to gut epithelium were also observed that might be associated with decreased survival of this insect.

**Conclusions:**

Our study reveals the toxic effects of *K. pneumoniae* and *P. paralactis* on biology of *S. litura*. These bacteria may be used as potential candidates for developing ecofriendly strategies to manage this insect pest.

## Introduction

*Spodoptera litura* (Fab.) (Lepidoptera: Noctuidae), commonly known as tobacco caterpillar, is one of the most destructive polyphagous pests. It feeds on a wide range of host plants belonging to more than 40 families. Cotton, alfalfa, berseem, maize, tobacco, groundnut, summer legumes, and vegetables like cucurbits, brinjal, potato, sweet potato etc. are among the most preferred host plants [1, 2]. Besides having high reproductive potential and strong migratory ability of adults, *S. litura* can adapt to wide range of ecological conditions. Thus under favourable conditions, its population increases in large numbers and causes economic losses to many of the commercially important crops [3–5]. The female lays eggs in masses, the early instar larvae feed gregariously while later instars spread and feed voraciously causing huge crop losses. The management of this pest is primarily relied on chemical insecticides and because of polyphagous nature; it has been exposed to a number of insecticides over the years. There are reports indicating development of varying levels of resistance in *S. litura* to different groups of insecticides such as pyrethroids, organophosphates, carbamates, abamectin, emamectin, benzoate, chlorantraniliprole and indoxacarb [6–10]. Moreover, these insecticides have potentially undesirable side effects on environment, humans and other non target species. Thus it becomes imperative to search for alternative ecofriendly strategies of pest management. Recently, considerable emphasis is being laid on the use of biopesticides based on microorganisms or their derivatives and plant products. Microbial pesticides based on fungi, bacteria, viruses and nematodes are gaining popularity due to their species specificity and environmental safety [11, 12].

Among the biocontrol agents, entomopathogenic bacteria and their toxins have been developed as commercial formulations which are being used successfully. Many *Bacillus* species viz. *B. popilliae*, *B. lentimorbus*, *B. larvae*, *B. thuringiensis*, *B. sphaericus* have been recognised as definitive insect pathogens [13, 14]. Apart from *Bacillus*, there are many other bacteria such as *Serratia*, *Photorhabdus*, *Xenorahabdus*, *Streptomyces* etc. which have also been reported as insect pathogens [15–18]. Among these, *B. thuringiensis* (Bt) is most successful and widely used against insect pests belonging to Diptera, Coleoptera and Lepidoptera. However, there are reports indicating development of resistance to Bt in lepidopteran pests viz. *Plutella xylostella* (Linnaeus), *Spodoptera frugiperda* (JE Smith), *Helicoverpa zea* (Boddie) and *Pectinophora gossypiella* (Saunders) [19–23]. This necessitates the search for more novel bacteria with insecticidal activity. Nowadays gut microbes isolated from insects have been explored for their insecticidal potential against agricultural pests [24, 25].

Insects are associated with a variety of microbes that play an important role in contributing nutrition, digestion, detoxification etc. [26, 27]. Gut microbiota particularly in termites and cockroaches help in digestion of cellulose while the aphids depend on gut microbes for their requirement of essential amino acids [28, 29]. Besides various beneficial roles, these gut bacteria may become opportunistic pathogens due to physiological or environmental changes that lead to perturbation in the gut microbial diversity [30, 31]. Various studies revealed the pathogenicity of enteric bacteria against insect hosts such as *Enterobacter cloacae* isolated from *S. litura* and *B. thuringiensis* isolated from *Spodoptera exigua* (Hubner) [24, 32]. *Flavobacterium* sp. and *Klebsiella* sp. isolated from *Spodoptera littoralis* (Boisduval) when tested for their virulence against same insect host exhibited 67% and 77% mortality respectively [33]. Similarly Sevim et al. [34] reported 60% mortality in *Agrotis segetum* (Denis & Schiffermuller) due to its gut bacteria *Enterococcus gallinarum*. Most of these entomopathogenic bacteria have been reported to produce diverse toxins with mode of action like *B. thuringiensis* [35, 36].

In order to develop ecologically sustainable strategies for pest control and to reduce the load of insecticides on environment, there is an increasing interest in finding indigenous bacterial isolates which are more pathogenic and effective against various insect pests. In this respect the present study aimed to explore the insecticidal potential of gut microbes isolated from adults of *S. litura.*

## Results

### Screening bioassays

**Fig. 1:**
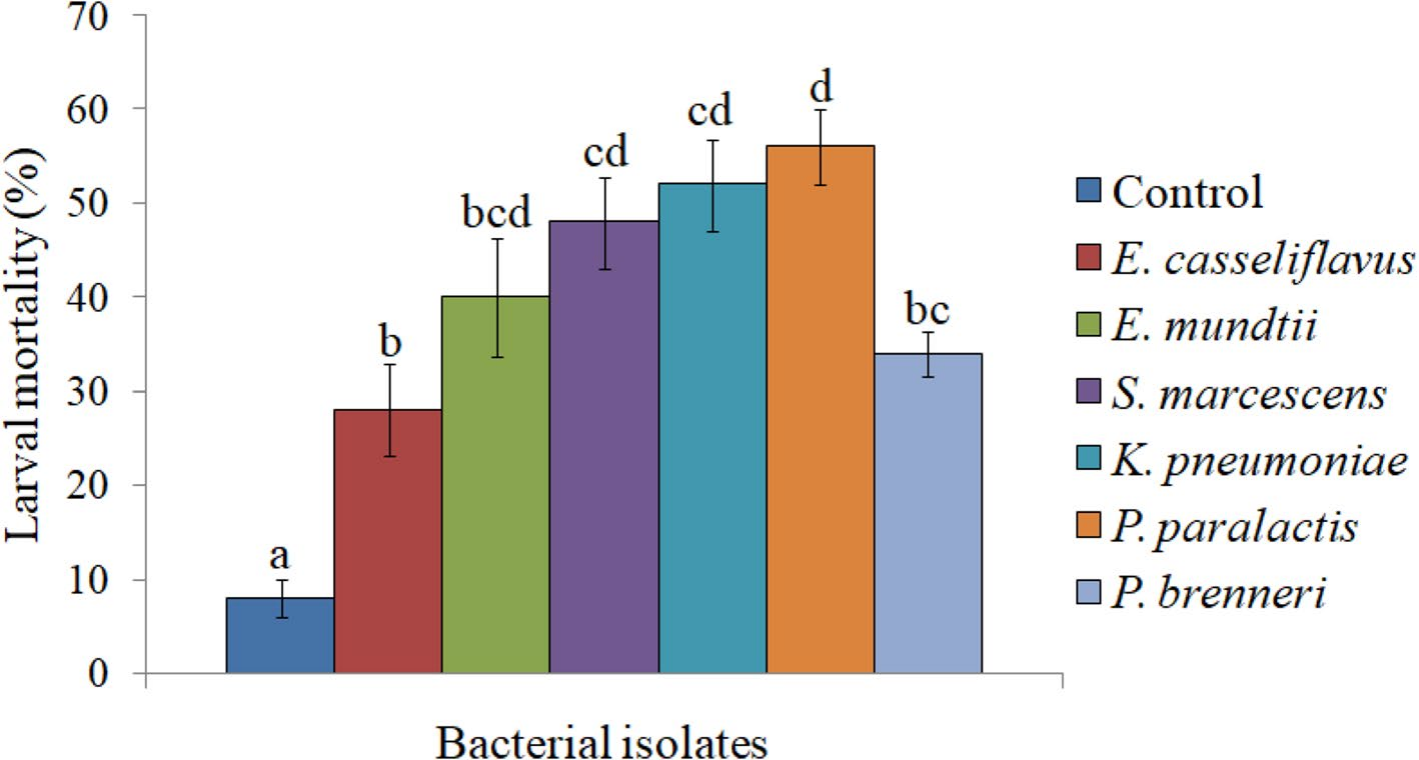
Pathogenicity of bacterial isolates of *S. litura *against its second-instar larvae at 1.8 × 10^9^cfu/ml (approx). Columns and bars represent the mean ± SE. Different letters above the columns representing each bacteria indicate significant differences at Tukey’s test *P* ≤ 0.05

A total of six bacteria i.e. *E. casseliflavus*, *E. mundtii*, *S. marcescens*, *K. pneumoniae*, *P. paralactis* and *P. brenneri* were isolated from adults of *S. litura*. Screening of these bacterial isolates exhibited varying level of virulence in *S. litura.* In comparison to control, all the bacterial treatments showed significantly high larval mortality (Fig. 1). Among these, *K. pneumoniae* and *P. paralactis* exhibited higher larval mortality i.e. 52% and 56% respectively, thus both were selected for detailed bioassay studies.

### Dose-response experiments

#### Mortality and development period

**Fig. 2:**
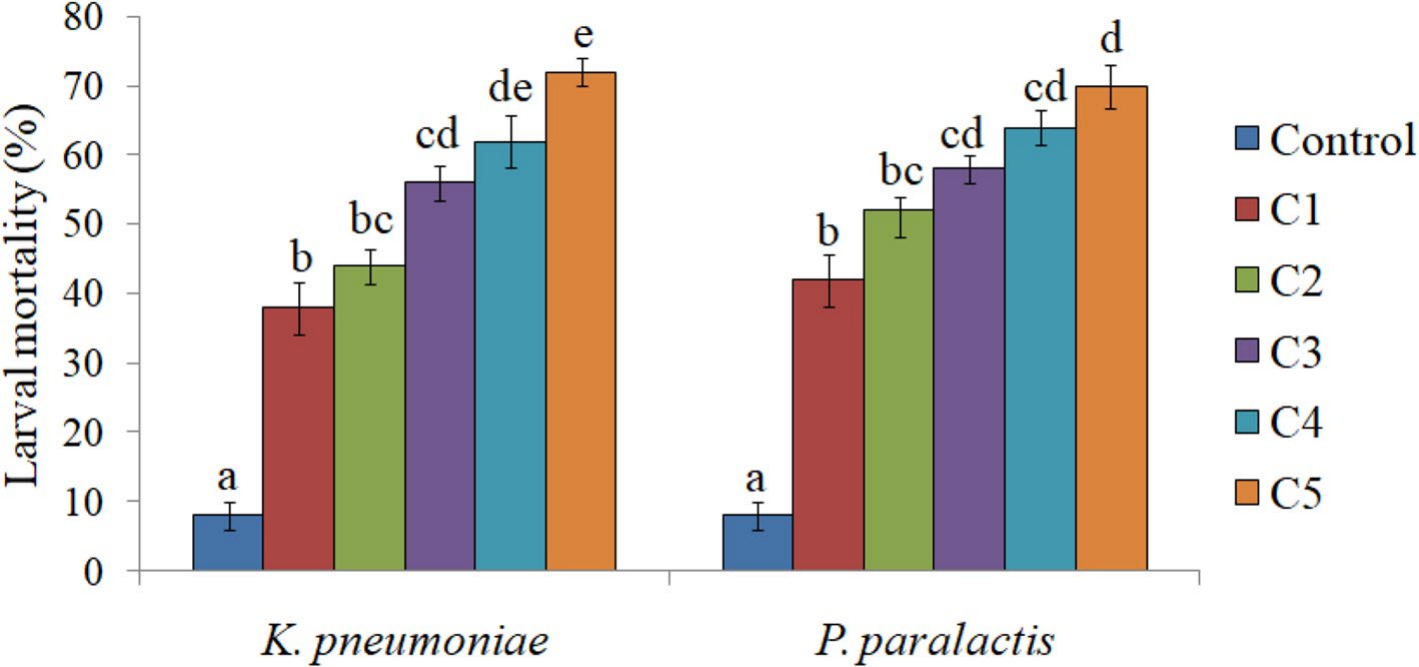
Effect of different concentrations of *K. pneumoniae *(C1=3.2 × 10^8^cfu/ml, C2=8.2 × 10^8^cfu/ml, C3=1.9 × 10^9^cfu/ml, C4= 3.6 × 10^9^cfu/ml and C5=5.8 × 10^9^cfu/ml) and *P. paralactis *(C1=2.4 × 10^8^cfu/ml, C2=6.8 × 10^8^cfu/ml, C3=1.4 × 10^9^cfu/ml, C4=3.2 × 10^9^cfu/ml and C5=5.0 × 10^9^cfu/ml) on larval mortality of *S. litura*. Columns and bars represent the mean ± SE. Different letters above the columns represent significant differences at Tukey’s test *P* ≤ 0.05

**Fig. 3:**
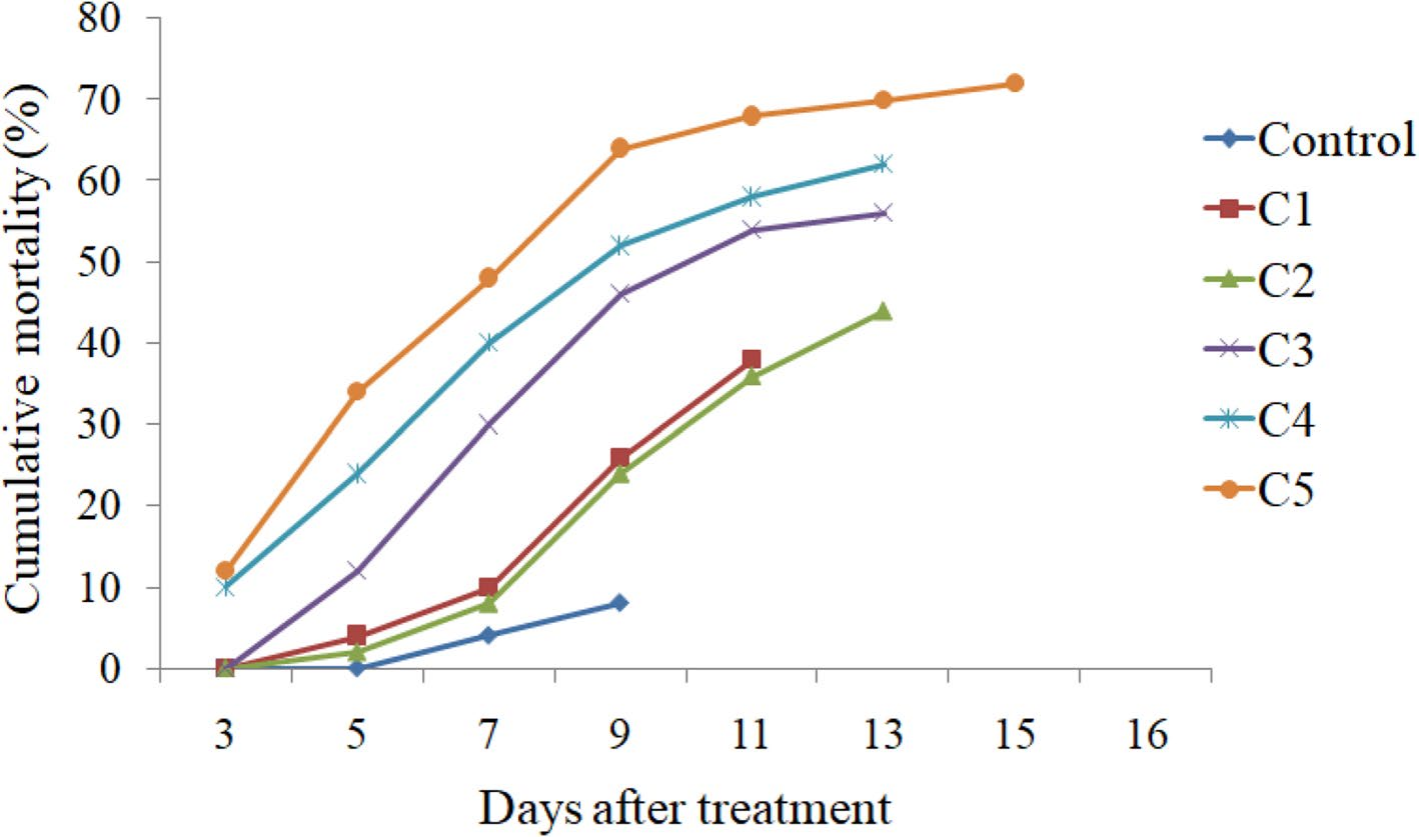
Mean cumulative mortality of second instar larvae of *S. litura *fed on castor leaves treated with different concentrations (C1=3.2 × 10^8^cfu/ml, C2=8.2 × 10^8^cfu/ml, C3=1.9 × 10^9^cfu/ml, C4=3.6 × 10^9^cfu/ml and C5=5.8 × 10^9^cfu/ml) of *K. pneumoniae*

**Fig. 4:**
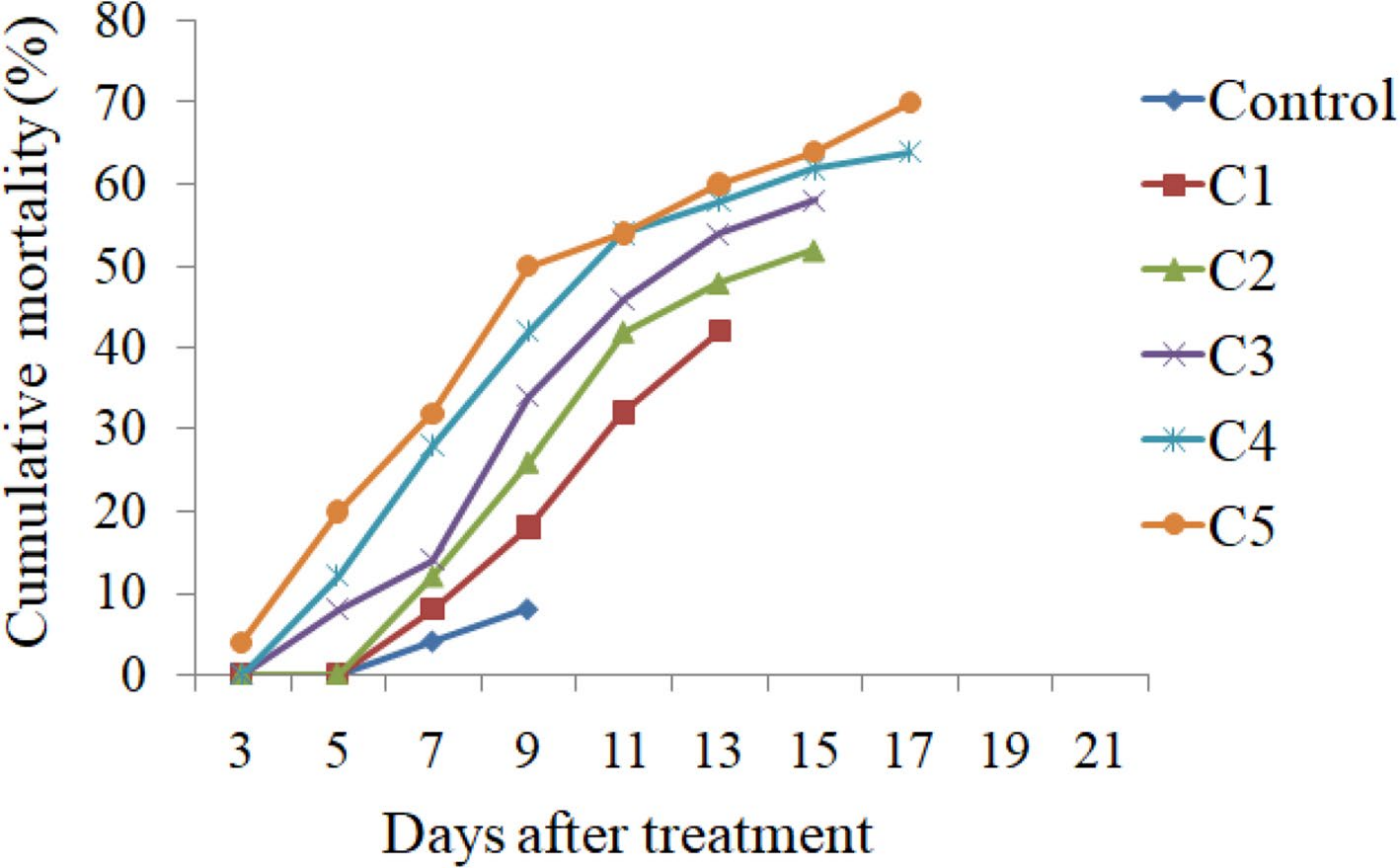
Mean cumulative mortality of second instar larvae of *S. litura *fed on castor leaves treated with different concentrations (C1=2.4 × 10^8^cfu/ml, C2=6.8 × 10^8^cfu/ml, C3=1.4 × 10^9^ cfu/ml, C4=3.2 × 10^9^ cfu/ml and C5=5.0 × 10^9^cfu/ml) of *P. paralactis*

**Fig. 5:**
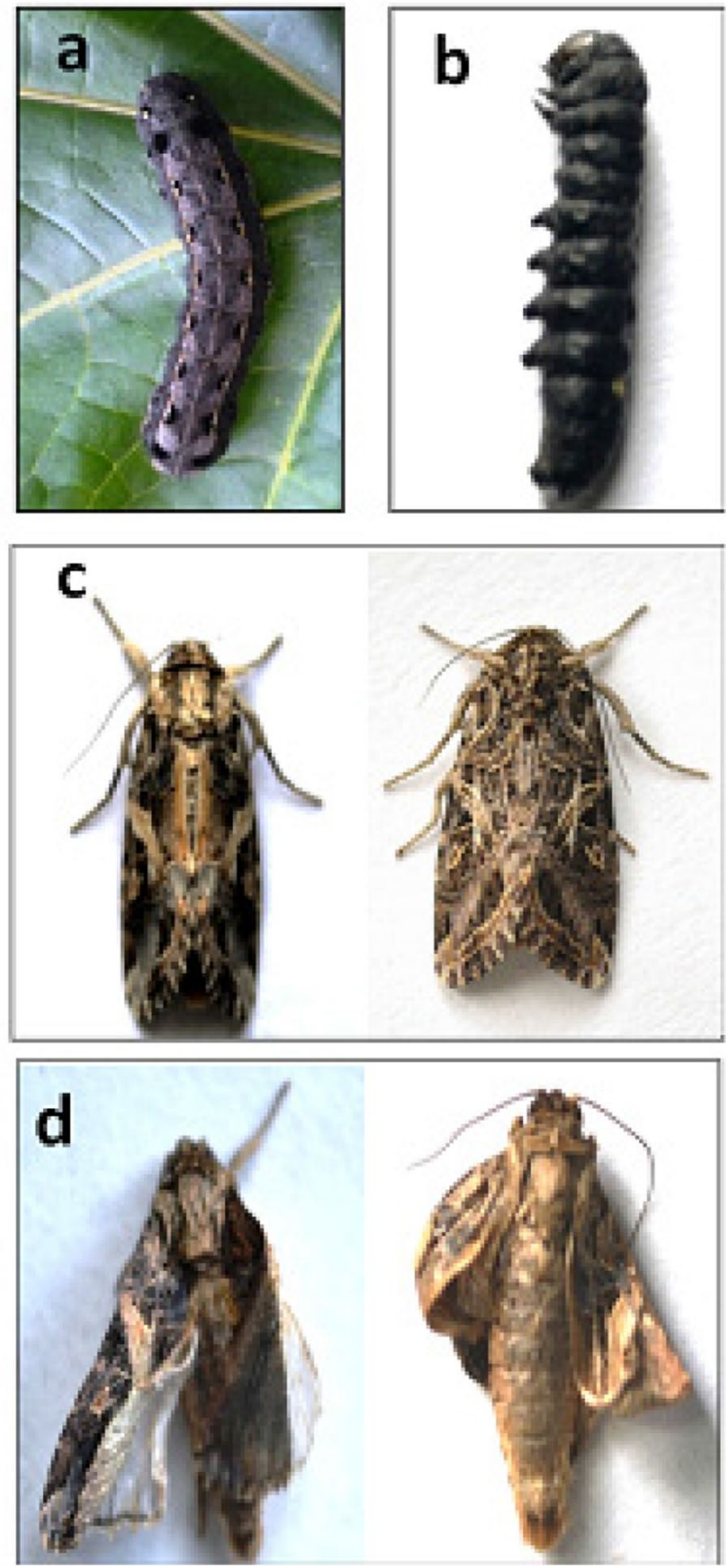
Effect of bacterial infection on *S. litura ***a** healthy larvae, **b** dead larvae, **c** normal adults and **d** morphologically deformed adults

A significant effect was observed on survival and development of *S. litura* when the larvae were fed on castor leaves treated with different concentrations of *K. pneumoniae* and *P. paralactis.* Both the bacteria caused significantly higher larval mortality in comparison to control. As is evident from Fig. 2, the mortality rate increased in a dose dependent manner. The larvae feeding on leaves treated with different concentrations of *K. pneumoniae* suffered 38.00-72.00% mortality (*F *=63.53, *p *≤ 0.05). Similarly *P. paralactis* caused 42.00-70.00% mortality in *S. litura* larvae (*F *=57.36, *p *≤0.05). The larval mortality started after third day of treatment at higher concentrations i.e. 3.6 × 10^9^ and 5.8 × 10^9^ cfu/ml of *K. pneumoniae* and at highest concentration (5.0 × 10^9^ cfu/ml) of *P. paralactis*. However, at lowest concentration the larval mortality started after five days in case of *K. pneumoniae* and seventh day after treatment in case of *P. paralactis* (Figs. 3 and 4). The LC_50_ values were calculated by using Probit analysis. It was found to be 1.2 × 10^9^ cfu/ml for *K. pneumoniae* and 6.4 × 10^8^ cfu/ml for *P. paralactis*. The infected larvae showed the symptoms of sluggishness, cessation of feeding and the dead larvae became black in color, flaccid with intact integument due to pathogenic effects of these bacteria (Fig. 5a and b).

Consumption of bacteria significantly delayed the development of insect (Table 1). Significant differences were observed among the treatments in case of larval development period. In comparison to control, the larvae took 2.38 to 4.74 days more to complete their development at 1.9 × 10^9^ to 5.8 × 10^9^ cfu/ml of *K. pneumoniae.* Significant effect was also detected on pupal development period. Except for the lowest concentration the total development period of *S. litura* extended significantly due to consumption of *K. pneumoniae*. Similarly *P. paralactis* influenced the development of *S. litura*. All the concentrations significantly delayed the larval development. With respect to control, the highest concentration i.e. 5.0 × 10^9^ cfu/ml extended the larval period by 8.04 days. Although no significant effect was observed on pupal period except for the highest concentration, but the total development period of *S. litura* was prolonged significantly at all the concentrations of *P. paralactis* (Table 1).

**Table 1:** Influence of different concentrations of *K. pneumoniae* and *P. paralactis* on development and adult emergence of *S. litura*

Bacteria	Concentrations (cfu/ml)	Larval period (days) (Mean± S.E.)	Pupal period (days) (Mean± S.E.)	Total developmental period (days) (Mean ± S.E.)	Adult emergence (%) (Mean± S.E.)	Adult deformities (%) (Mean± S.E.)
***K. pneumoniae***	Control	12.06 ± 0.48^a^	8.90 ± 0.19^a^	20.96 ± 0.40^a^	91.06 ± 4.17 ^b^	3.20 ± 0.80^a^
	3.2 × 10^8^	12.47 ± 0.66^ab^	9.60 ± 0.18^ab^	22.07 ± 0.59^ab^	81.80 ± 2.26^ab^	8.40 ± 0.74^a^
	8.2 × 10^8^	14.10 ± 0.43^ab^	10.03 ± 0.40^ab^	24.13 ± 0.69^bc^	78.80 ± 2.08^ab^	9.00 ± 1.94^a^
	1.9 × 10^9^	14.44 ± 0.38^bc^	10.55 ± 0.40^b^	24.99 ± 0.48^ cd^	77.80 ± 4.59^ab^	10.20 ± 2.65^ab^
	3.6 × 10^9^	16.30 ± 0.48^ cd^	10.60 ± 0.36^b^	26.90 ± 0.64^d^	74.60 ± 2.01^a^	19.00 ± 2.38^bc^
	5.8 × 10^9^	16.80 ± 0.48^d^	10.70 ± 0.20^b^	27.50 ± 0.63^d^	70.52 ± 2.53^a^	22.00 ± 3.52^c^
	F-value	14.98**	5.16**	19.69**	5.04**	10.01**
***P. paralactis***	Control	12.06 ± 0.48^a^	8.90 ± 0.19^a^	20.96 ± 0.40^a^	91.06 ± 4.17^c^	3.20 ± 0.80^a^
	2.4 × 10^8^	14.88 ± 0.84^b^	8.90 ± 0.45^a^	23.78 ± 0.71^b^	84.20 ± 1.35^bc^	10.20 ± 0.66^ab^
	6.8 × 10^8^	16.16 ± 0.28^b^	9.36 ± 0.18^ab^	25.52 ± 0.22^bc^	79.60 ± 1.16^abc^	14.60 ± 1.32^abc^
	1.4 × 10^9^	17.09 ± 0.34^bc^	9.54 ± 0.16^ab^	26.63 ± 0.47^ cd^	72.60 ± 3.35^ab^	23.60 ± 5.50^bc^
	3.2 × 10^9^	18.90 ± 0.50^ cd^	9.70 ± 0.12^ab^	28.60 ± 0.50^de^	72.60 ± 2.76^ab^	24.80 ± 5.00^bc^
	5.0 × 10^9^	20.10 ± 0.64^d^	10.40 ± 0.24^b^	30.50 ± 0.67^e^	71.00 ± 1.78^a^	28.00 ± 5.83^c^
	F-value	27.46**	4.97**	42.05**	8.90**	6.08**

The values (Mean ± SE) followed by different letters (superscript) with in a column indicate the significant differences at Tukey’s test *P* ≤ 0.05, **Significant at 1% level

#### Adult emergence and reproductive potential

**Fig. 6:**
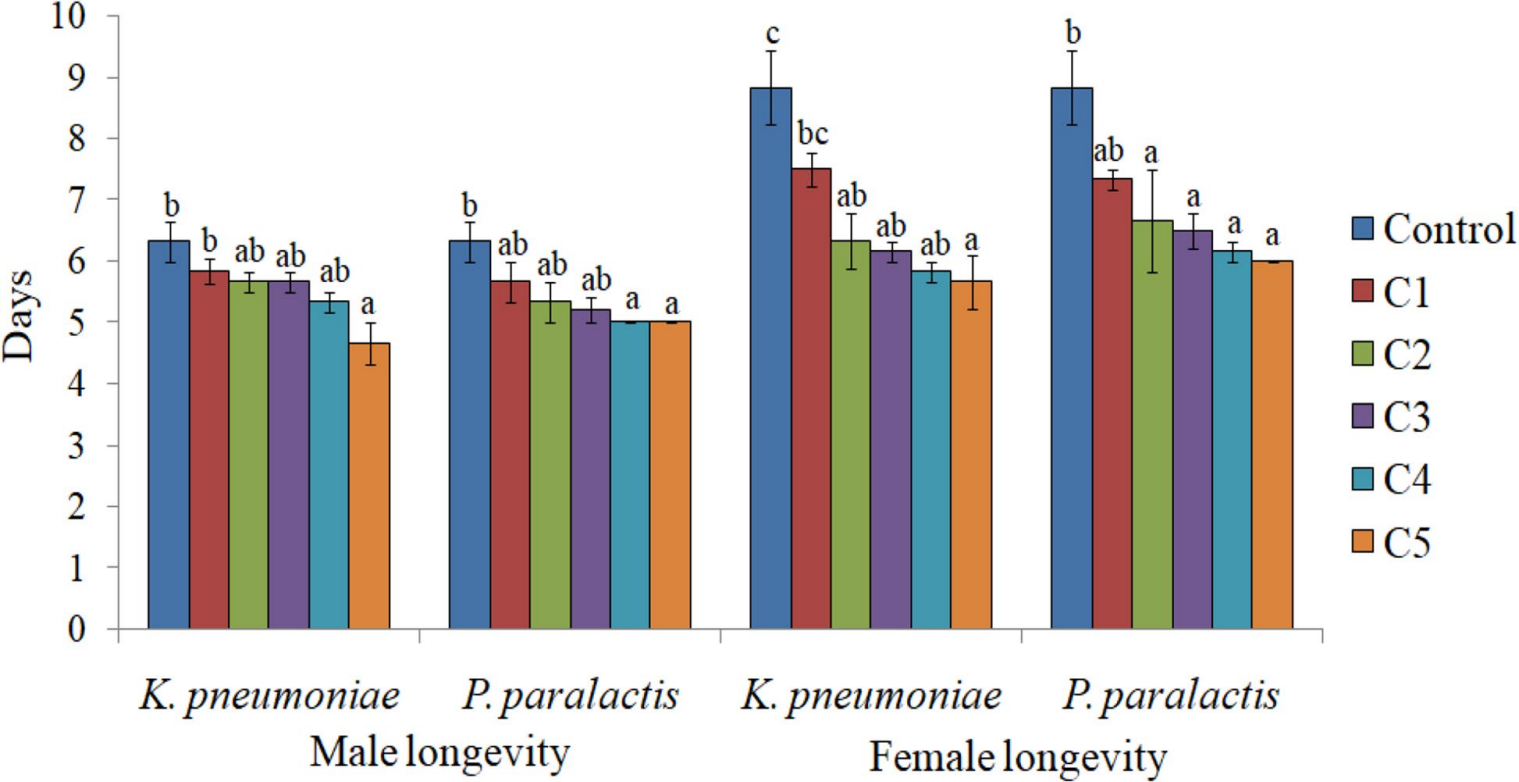
Influence of different concentrations of *K. pneumoniae *(C1 = 3.2 × 10^8^ cfu/ml, C2 =8.2 × 10^8^cfu/ml, C3 =1.9 × 10^9^cfu/ml, C4 = 3.6 × 10^9^ cfu/ml and C5 =5.8 × 10^9^ cfu/ml) and *P. paralactis* (C1 =2.4 × 10^8^cfu/ml, C2 =6.8 × 10^8^ cfu/ml, C3 =1.4 × 10^9^ cfu/ml, C4 =3.2 × 10^9^ cfu/ml and C5 =5.0 × 10^9^ cfu/ml) on adult longevity of *S. litura*. Columns and bars represent the mean ± SE. Different letters above the columns represent significant differences at Tukey’s test *P* ≤ 0.05

**Fig. 7:**
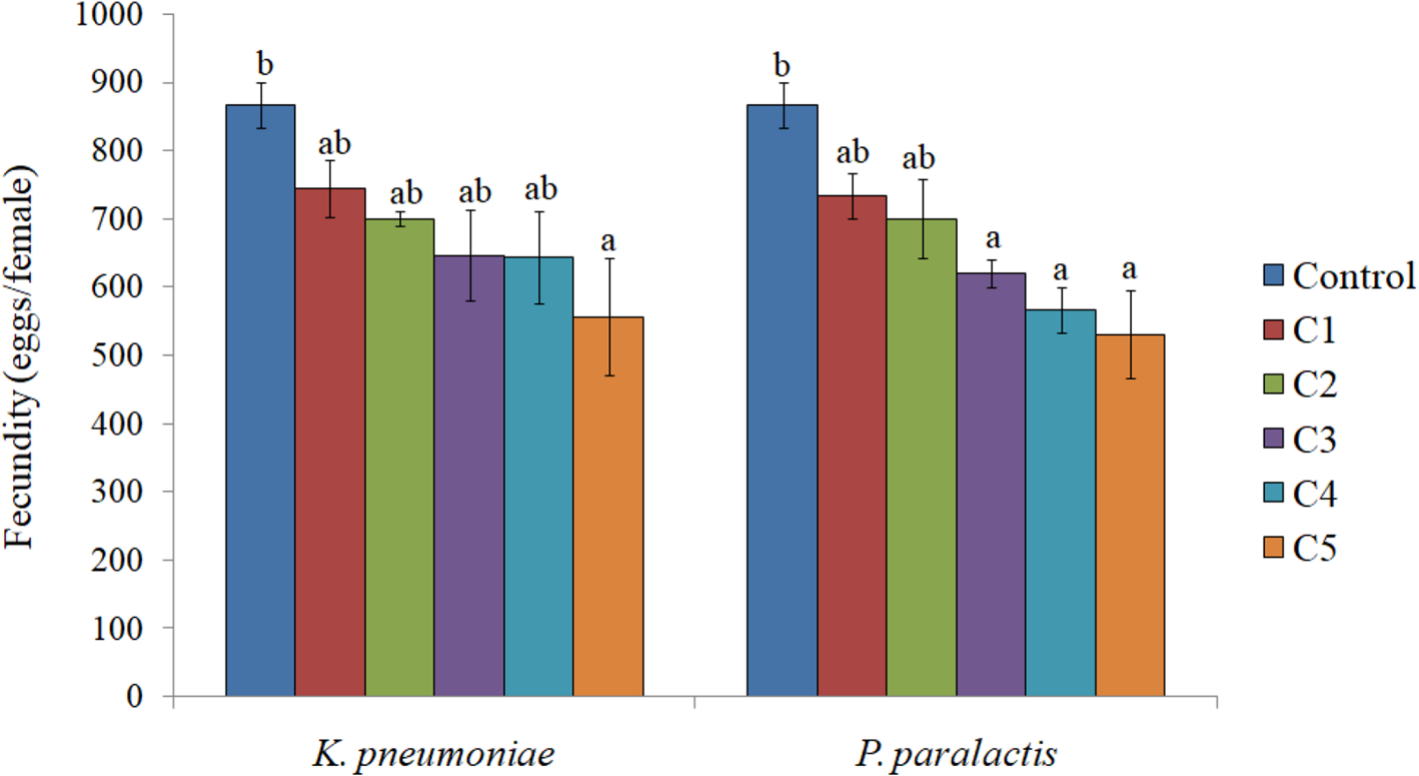
Influence of different concentrations of *K. pneumoniae *(C1 = 3.2 × 10^8^ cfu/ml, C2 =8.2 × 10^8^ cfu/ml, C3 =1.9 × 10^9^cfu/ml , C4 = 3.6 × 10^9^ cfu/ml and C5 =5.8 × 10^9^ cfu/ml) and *P. paralactis *(C1 =2.4 × 10^8^ cfu/ml, C2 =6.8 × 10^8^ cfu/ml, C3 =1.4 × 10^9^cfu/ml, C4 =3.2 × 10^9^cfu/ml and C5 =5.0 × 10^9^ cfu/ml) on fecundity of *S. litura*. Columns and bars represent the mean ± SE. Different letters above the columns represent significant differences at Tukey’s test*P* ≤ 0.05

**Fig. 8:**
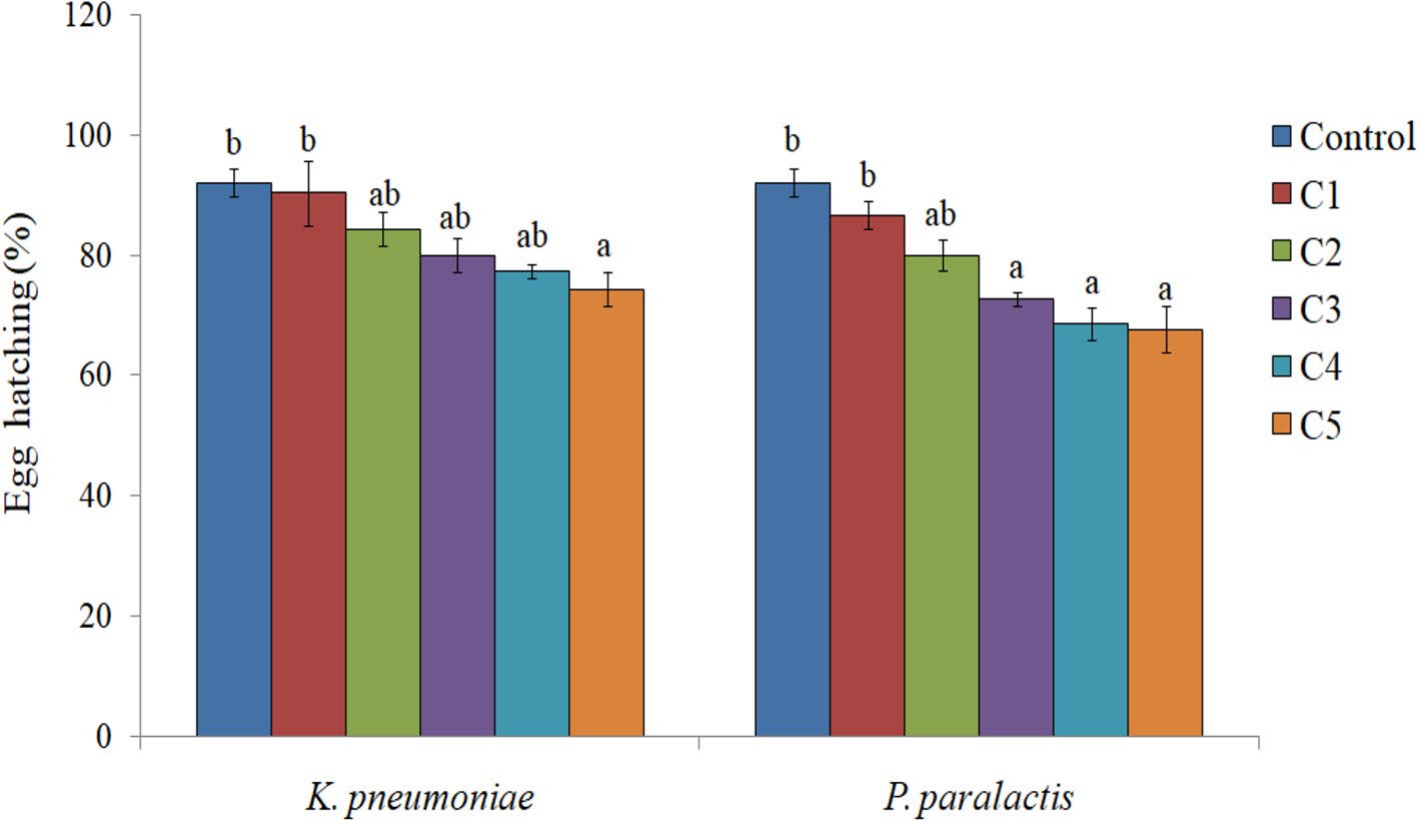
Influence of different concentrations of *K.pneumoniae *(C1 = 3.2 × 10^8^ cfu/ml, C2 =8.2 × 10^8^ cfu/ml, C3 =1.9 × 10^9^cfu/ml , C4 = 3.6 × 10^9^ cfu/ml and C5 =5.8 × 10^9^ cfu/ml) and *P. paralactis* (C1 =2.4 × 10^8^cfu/ml, C2 =6.8 × 10^8^ cfu/ml, C3 =1.4 × 10^9^ cfu/ml, C4 =3.2 × 10^9^cfu/ml and C5 =5.0 × 10^9^ cfu/ml) on egg hatching of *S. litura*. Columns and bars represent the mean ± SE. Different letters above the columns represent significant differences at Tukey’s test *P* ≤ 0.05

Higher concentrations of both the bacteria significantly decreased the emergence of adults. In comparison to 91.06% in control, only 70.52 and 71.00% adult emergence was recorded due to *K. pneumoniae* and *P. paralactis* at 5.8 × 10^9^ cfu/ml and 5.0 × 10^9^ cfu/ml respectively (Table 1). The adults emerged from treated larvae exhibited morphological deformities such as unequal and crumpled wings (Fig. 5c and d). As is evident from Fig. 6, both the bacterial treatments also influenced the adult longevity with significant effect at higher concentrations. Fecundity of females tended to decrease under the influence of *K. pneumoniae*, but significant effect was only detected at the highest concentration where the female laid only 556 eggs during its lifetime in comparison to 866.66 eggs/female in control. Higher concentrations of *P. paralactis* also significantly decreased the fecundity (Fig. 7). Viability of eggs was also adversely affected at higher concentrations of the bacterial cell suspensions (Fig. 8).

#### Effect of *K. pneumoniae* and *P. paralactis* on nutritional physiology

The results presented in Table 2, depict reduction in RCR of *S. litura* larvae feeding on leaves treated with different concentrations of cell suspension of both the bacteria when compared to control. The differences were statistically significant at higher concentrations. Decrease in consumption rate further lead to decrease in relative growth rate of larvae. With respect to control there was 30.30 to 33.33% and 24.24 to 36.36% decrease in RGR due to different concentrations of *K. pneumoniae* and *P. paralactis* respectively (*F *=5.19, *p *≤0.05; *F* =4.88, *p *≤0.05). Ingestion of leaves treated with both the bacteria also influenced the efficiency of conversion of ingested and digested food of *S. litura*. The higher concentrations of *K. pneumoniae* cell suspension resulted in 2.04 to 2.06 times decrease in ECI and 1.79 to 1.80 times decrease in ECD with respect to control. As is evident from Table 2, all the concentrations of *P. paralactis* also significantly decreased the values of ECI and ECD with respect to control. However, statistically significant differences were not observed among the concentrations. Higher concentrations of *K. pneumoniae* significantly decreased the approximate digestibility while no significant effect was detected due to *P. paralactis* except for the highest concentration.

**Table 2:** Influence of different concentrations of *K. pneumoniae* and *P. paralactis* on food consumption and utilization of *S. litura* larvae

Bacteria	Concentrations (cfu/ml)	RGR (mg mg^−1^d^−1^) (Mean± S.E.)	RCR (mg mg^−1^d^−1^) (Mean± S.E.)	ECI (%) (Mean± S.E.)	ECD (%) (Mean± S.E.)	AD (%) (Mean± S.E.)
***K. pneumoniae***	Control	0.33 ± 0.040^b^	34.56 ± 0.83^c^	1.82 ± 0.24^b^	8.04 ± 0.86^b^	95.51 ± 1.28^c^
	3.2 × 10^8^	0.23 ± 0.008^a^	29.02 ± 1.88^bc^	1.50 ± 0.19^ab^	8.33 ± 0.93^b^	91.61 ± 0.37^bc^
	8.2 × 10^8^	0.25 ± 0.006^a^	27.02 ± 1.01^bc^	1.31 ± 0.18^ab^	6.53 ± 0.46^ab^	91.39 ± 0.81^bc^
	1.9 × 10^9^	0.23 ± 0.01^a^	18.09 ± 4.26^ab^	1.24 ± 0.15^ab^	4.47 ± 0.67^a^	90.32 ± 1.21^b^
	3.6 × 10^9^	0.25 ± 0.002^a^	13.58 ± 4.32^a^	0.89 ± 0.06^a^	4.45 ± 0.57^a^	87.18 ± 0.70^ab^
	5.8 × 10^9^	0.22 ± 0.01^a^	10.51 ± 3.50^a^	0.88 ± 0.05^a^	4.45 ± 0.57^a^	84.66 ± 1.70^a^
	F-value	5.19**	9.90**	4.73**	6.90**	11.69**
***P. paralactis***	Control	0.33 ± 0.040^b^	34.56 ± 0.83^c^	1.82 ± 0.24^b^	8.04 ± 0.86^b^	95.51 ± 1.28^b^
	2.4 × 10^8^	0.25 ± 0.010^a^	33.19 ± 0.61^bc^	0.83 ± 0.02^a^	4.15 ± 0.72^a^	91.76 ± 1.44^ab^
	6.8 × 10^8^	0.25 ± 0.006^a^	29.02 ± 1.88^ab^	0.88 ± 0.05^a^	4.45 ± 0.57^a^	91.39 ± 0.81^ab^
	1.4 × 10^9^	0.25 ± 0.005^a^	29.15 ± 0.84^ab^	0.88 ± 0.01^a^	4.49 ± 0.31^a^	91.69 ± 0.65^ab^
	3.2 × 10^9^	0.23 ± 0.017^a^	27.02 ± 1.01^a^	0.80 ± 0.04^a^	4.47 ± 0.67^a^	90.12 ± 1.17^ab^
	5.0 × 10^9^	0.21 ± 0.010^a^	27.55 ± 1.23^a^	0.76 ± 0.02^a^	3.75 ± 0.39^a^	84.74 ± 3.57^a^
	F-value	4.88**	7.22**	14.95**	6.39**	3.88**

The values (Mean ± SE) followed by different letters (superscript) with in a column indicate the significant differences at Tukey’s test *P* ≤ 0.05, **Significant at 1% level

#### Effect of *K. pneumoniae* and *P. paralactis* on gut microflora of *S. litura*

Gut microbial diversity of control as well as larvae treated with bacterial suspension of *K. pneumoniae* and *P. paralactis* at their LC_50_ values i.e. 1.2 × 10^9^ and 6.4 × 10^8^ cfu/ml respectively was explored in order to see the difference in culturable bacteria. Gut microbial composition differed in control and treated larvae. *E. mundtii*, *E.casseliflavus* and *A. hemolyticus* were isolated from control larvae having cfu count of 7.4 × 10^6^, 6.9 × 10^6^ and 4.0 × 10^5^ per ml respectively (Table 3). However, when the larvae were fed on castor leaves treated with *K. pneumoniae*, three cultures i.e. *E. mundtii*, *E. casseliflavus* and *K. pneumoniae* were present with 7.0 × 10^5^, 7.5 × 10^4^ and 8.2 × 10^7^ cfu/ml respectively (Table 3). Similarly *E. mundtii*, *E. casseliflavus* and *P. paralactis* were present with 7.3 × 10^4^, 4.2 × 10^3^ and 6.6 × 10^6^ cfu/ml respectively when the larvae were fed on *P. paralactis* treated leaves. However, *A. hemolyticus* was absent in the larvae treated with both the bacterial concentrations. The numbers of *Enterococcus* colonies were superseded by the number of colonies of *K. pneumoniae* and *P. paralactis* in the treated larvae.

**Table 3:** Effect of LC_50_ values of *K. pneumoniae* and *P. paralactis* on culturable gut microbial diversity of *S. litura*

Treatments	Bacterial population in gut of *S. litura* larvae (cfu/ml)				
	*E. mundtii*	*E. casseliflavus*	*K. pneumoniae*	*P. paralactis*	*A.hemolyticus*
Control	7.4 × 10^6^	6.9 × 10^6^	-	-	4.0 × 10^5^
* K. pneumoniae*	7.0 × 10^5^	7.5 × 10^4^	8.2 × 10^7^	-	-
* P. paralactis*	7.3 × 10^4^	4.2 × 10^3^	-	6.6 × 10^6^	-

### Histological studies

**Fig. 9:**
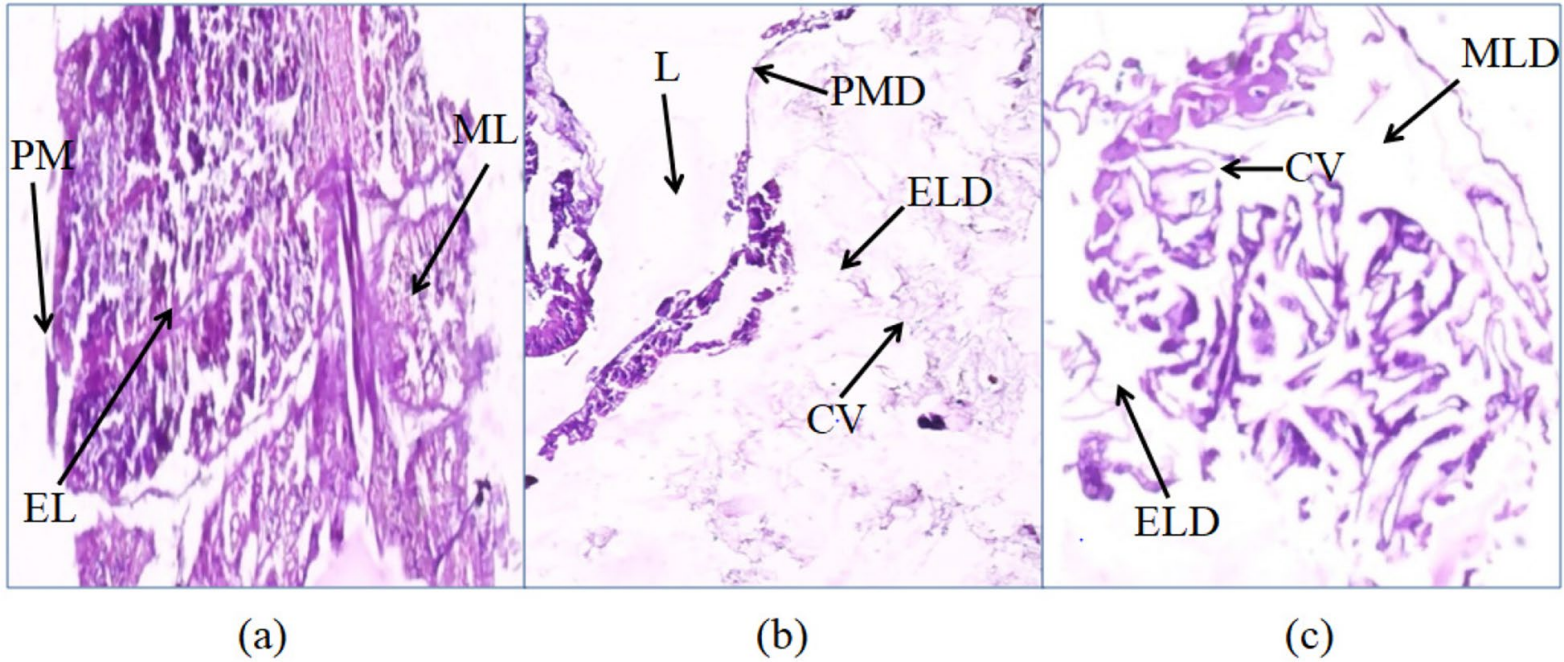
Longitudinal section through the midgut of 4th instar *S. litura* larvae **a** midgut of larvae fed on control leaf diet showing intact peritrophic membrane (PM), epithelial layers (EL) and muscle layer (ML), **b** midgut of larvae fed on leaf treated with *K. pneumoniae* showing lumen (L), peritrophic membrane disruption (PMD), epithelial layers disruption (ELD) and cytoplasmic vacuolation (CV), **c** midgut of larvae fed on leaf treated with *P. paralactis *showing cytoplasmic vacuolation (CV), epithelial layers disruption (ELD) and muscle layer disruption (MLD)

The histopathological effects of *K. pneumoniae* and *P. paralactis* on the midgut of *S. litura* larvae were also detected. The midgut cross-sections of treated larvae showed damage of the midgut epithelial cells (Fig. 9). Midgut of larvae fed with *K. pneumoniae* and *P. paralactis* showed the vacuolization of the cytoplasm, brush border membrane destruction and complete destruction of membrane at some sites. In contrast, the control *S. litura* larvae showed a well-preserved layer of epithelial cells with unaffected apical microvilli membrane of the midgut.

### Evaluation of the presence of *K. pneumoniae* and *P. paralactis* in larval haemolymph

The growth of *K. pneumoniae* bacteria was observed in the hemolymph of larvae infected with *K. pneumoniae*, however, no growth was observed in case of *P. paralactis* treatment as well as in control larvae.

## Discussion

Insects live in a symbiotic relationship with various gut microbes that play an important role in nutrition and digestion, development, detoxification of secondary plant metabolites and reproduction of insects [26, 27, 37]. In the present study culturable bacteria viz. *E. casseliflavus*, *E. mundtii*, *S. marcescens*, *K. pneumoniae*, *P. paralactis* and *P. brenneri* were isolated from the gut of adults of *S. litura*. These bacteria have earlier been reported to be associated with larvae and adults of lepidopterans and other insects [38–40]. In order to identify new candidates for biological control, these bacteria were tested for their effect on survival and development of *S. litura.* Among these *K. pneumoniae* and *P. paralactis* induced up to 72 and 70% mortality respectively in the larvae. Pathogenicity of *K. pneumoniae* and *Pseudomonas* species have earlier been reported against the same host as well as other insects. *Klebsiella* sp. isolated from *S. littoralis* and *Bombyx mori* (Linnaeus) showed high insecticidal activity when tested against the same host [33, 41]. The pathogenicity of different strains of *K. pneumoniae* has also been documented against *Galleria mellonella* (Linnaeus) causing 100% mortality when the larvae were injected with highest concentration (10^7^ cfu) after 24 h of infection [42]. *K. pneumoniae* isolated from infected pupae of *G. mellonella* also caused cross pathogenicity in *Trichoplusia ni* (Hubner) larvae [40].

*Pseudomonas* is a broad-host-range entomopathogenic bacterium that exhibits insecticidal activity towards agricultural pests. *Pseudomonas* strains have been found to infect and kill larval stages of *Drosophila melanogaster* (Meigen), *S. littoralis* and *P. xylostella* [36, 43, 44]. Maciel-Vergara et al. [45] documented higher mortality in the larvae of the giant mealworm *Zophobas morio* (Fabricius) due to *P*. *aeruginosa* when injected into the hemocoel in comparison to oral ingestion. Other species of *Pseudomonas* viz. *P. taiwanensis*, *P. protegens* strains and *P. chlororaphis* have also been reported to have potent insecticidal activity against *G. mellonella* and *Manduca sexta* (Linnaeus) larvae [36, 46]. Contrary to this, there are reports indicating the role of these bacteria in fitness of insect. *Klebsiella oxytoca* helps to reduce the intraspecific competition in age-disparate larval offsprings by affecting the ovipositional behaviour of gravid females of *Musca domestica* (Linnaeus) [47]. Previous studies also documented the role of *Klebsiella* spp. in increasing the mating performance in *Ceratitis capitata* (Wiedemann) [48–50]. Similarly *Pseudomonas* genus has been reported to help in digestion of cellulose, amino acid synthesis and production of siderophores for extraction of iron required in many biochemical reactions, and to overcome iron toxicosis [51–53].

The larvae treated with *K. pneumoniae* and *P. paralactis* showed the symptoms of sluggishness, cessation of feeding and the dead larvae became black in color, flaccid with intact integument. Similar symptoms have earlier been reported in other insects due to *Klebsiella* and *Pseudomonas* infection [42, 45]. Histopathological studies revealed disruption of peritrophic matrix, damage in microvilli and midgut epithelial cells of *S. litura* larvae due to *K. pneumoniae* and *P. paralactis* infection. Damage to peritrophic membrane and disruption of intestinal integrity has earlier been reported due to oral ingestion of *Pseudomonas* and Bt toxins [44, 54, 55]. The pathogenicity of bacteria may be due to toxemia or septicemia. The larvae treated with *P. paralactis* did not show any bacterial growth in the hemolymph, indicating that the mortality of larvae may be due to toxin production and not due to its entry into the hemocoel. However, *K. pneumoniae* had been found to cross the gut epithelial barrier and invade the hemocoel causing septicaemia and ultimately death of the host. Previously Insua et al. [42] documented mortality in *G. mellonella* due to replication of *K. pneumoniae* in hemocoel. The high bacterial load present in the hemolymph cause concomitant tissue necrosis due to bacterial toxins [56, 57]. *Pseudomonas* is known to produce toxins such as extracellular proteinases and metalloproteases causing larval mortality in insects [45, 58]. The gut bacteria persist usually in low numbers inside the insect host without causing any disease; however, they may become pathogenic under stress conditions when the insect immune system gets weakened or due to alterations in the composition of microbiota [59–61]. *S. marcescens*, *Pseudomonas* and *klebsiella* are present as a part of normal gut microflora of lepidopterans and other insects [39, 62, 63]. However, the pathogenicity of these bacteria isolated from insects has also been reported against same host as well as other insects [33, 45, 64]. Present study reveals the change in gut microbial diversity between the infected and control larvae. The composition of gut microflora of control larvae consists of three cultures viz. *E. mundtii*, *E. casseliflavus* and *A. hemolyticus* in uniform distribution. However, in case of larvae fed on diet treated with *K. pneumoniae* and *P. paralactis*, the number of respective bacteria increased in comparison to *E. mundtii* and *E. casseliflavus* and thus become dominant in infected larvae and inhibited the growth of *A. hemolyticus*. Similar reports have earlier been documented by Thakur et al. [32] and Broderick et al. [61] in lepidopterans pests. Perturbation in gut microbial diversity due to bacterial infection thus lead to death of the host [60, 65–67].

In addition to mortality, larval treatment with *K. pneumoniae* and *P. paralactis* also delayed the development period of *S. litura.* It is in line with the previous studies indicating delay in development of *S. litura* and *Bactrocera dorsalis* (Hendel) due to infection of gut bacteria *Enterobacter cloacae* and *Lactobacillus lactis* [32, 68]. The results also revealed a significant negative effect of *K. pneumoniae* and *P. paralactis* on nutritional parameters of *S. litura.* The relative consumption rate of *S. litura* significantly decreased when the larvae were fed on diet treated with higher concentrations of bacterial cell suspension. Decreased RCR further led to concomitant decrease in growth rate relative to control. The treated larvae also showed reduction in efficiency of conversion of ingested and digested food and approximate digestibility of insect. Similar inhibitory effects of bacterial infection on nutritional physiology have earlier been documented on *S. litura* and *Cnaphalocrocis medinalis* (Guenee) [32, 69]. The decrease in consumption rate may be due to antifeedant effect of bacteria which impairs the larva from feeding or prohibits it from making maximum utilization of the ingested diet that may lead to longer larval developmental time [68]. The bacterial infection also caused damage to epithelial membrane and peritrophic matrix which may further interrupt the digestion and nutrient absorption thereby slowing the growth of larvae as suggested by Buchon et al. [70]. The *Pseudomonas* bacteria are known to produce chitinases that hydrolyse chitin, which is a common constituent of the insect exoskeleton and midgut peritrophic membrane [71–73]. Chitinases have been reported to disrupt the peritrophic membrane and decrease the digestive function [74–76]. An extracellular chitinase purified from *B. subtilis* has been found to negatively affect the nutritional parameters of *S. litura* [77]. The reduced adult emergence and morphological deformities in adults such as unequal and crumpled wings were observed along with the decrease in reproductive potential of adults. Olcott et al. [43] also documented delay in development as well as morphological defects in adult flies of *D. melanogaster* due to *P. fluorescens* infection. Similarly *P. aeruginosa* infection negatively affected the longevity of *C. capitata* [38]. These results show that the bacteria isolated from *S. litura* act as opportunistic pathogens which exert growth inhibition, antifeedant and toxic effects on *S. litura.*

## Conclusions

In conclusion, cultivable bacteria viz. *K. pneumoniae* and *P. paralactis* isolated from the gut of *S. litura* adults exhibited insecticidal potential. Both these bacteria caused significantly higher mortality in *S. litura* larvae and delayed the development of insect. These bacteria also negatively affected the nutritional physiology and reproductive potential of the insect. Thus both these bacterial isolates appear to be significant candidates for microbial control of this pest. However, further optimization studies on mass production of bacterial cells and their testing under natural conditions need to be done.

## Materials and methods

### Collection and mass rearing of insect

The egg masses and larvae of *S. litura* were collected from cabbage and cauliflower fields around Amritsar (Punjab), India. The larvae were reared in the laboratory on fresh castor leaves in plastic jars (15 cm×10 cm) under controlled temperature and humidity conditions of 25 ± 2^o^C and 60 ± 5% respectively [53]. The larval diet was changed daily till pupation. The pupae were transferred to pupation jars and freshly emerged adults were shifted to oviposition jars. The adults were provided with honey solution (1 part honey: 4 parts water v/v) soaked on a cotton swab. The oviposition jars were lined with filter paper to facilitate egg laying. The newly hatched larvae were transferred to fresh castor leaves for further maintenance of culture. Larvae from the laboratory culture were used for conducting experiments.

### Bacterial isolation

The male and female adults (4 days old) obtained from laboratory culture were surface sterilised by rinsing with sterile water followed by 70% (v/v) ethanol, and then thoroughly rinsed with sterilized distilled water to remove the disinfectant. The adults were dissected aseptically with the help of sterilized micro scissors to remove the gut. The dissected guts of male and female adults were then separately homogenized in 1.0 ml Phosphate Buffer Saline (PBS) solution (pH 7.0). Homogenised samples were then serially diluted up to ten times and 100 µl of each diluted sample was then plated on Luria Bertani (LB) plates for isolation of bacteria. The plating was done by spread plate technique. The whole procedure was carried out under the laminar flow cabinet (ESCO, USA). The plates were incubated at 30 °C for 72 h and the morphologically distinct isolates were obtained. The pure bacterial cultures were stored in 50% (w/v) glycerol at -80 °C. Microbial isolates were identified as *Enterococcus casseliflavus*, *Enterococcus mundtii*, *Serratia marcescens*, *Klebsiella pneumoniae*, *Pseudomonas paralactis* and *Pantoea brenneri* (data submitted elsewhere). All these bacterial isolates were present in the females while the males harboured only *E. casseliflavus*, *E. mundtii*, *S. marcescens* and *K. pneumoniae.*

### Preparation of bacterial suspension

Different bacterial cultures were inoculated into LB broth and incubated at 30 °C for 48 h. After incubation each bacterial culture was centrifuged at 4000 rpm and 4˚C for 10 min to obtain the pellet. The pellet was washed once with sterile distilled water and resuspended in PBS. The bacterial density was measured at optical density (OD_600_) and adjusted to 1.89 (1.8 × 10^9^ cfu/ml approximately) and 10 ml of adjusted culture was further used in bioassays as described by Eski et al. [24] with some modifications.

### Screening bioassays

The screening of bacterial cultures for their insecticidal potential was conducted on second instar larvae (6 days old) of *S. litura*. The larvae were randomly selected and kept in rearing vials. Fresh castor leaves were surface sterilized with 5% (v/v) NaOCl followed by washing with distilled water. These leaves (approximately 10 cm^2^) were treated by dipping in 10 ml bacterial cell suspension of different isolates prepared as described above. The treated leaves after air drying at room temperature were placed in rearing vials containing larvae. In order to avoid cannibalism, one larva was kept in each rearing tube. Initial screening was done with 50 larvae with 5 replications of each bacterial isolate (10 larvae per replicate). Surface sterilized castor leaves dipped in PBS buffer were fed to control group. The experimental conditions were maintained at 25 ± 2^o^C temperature and 60 ± 5% relative humidity. The diet was changed after every 48 h and for that larvae were provided with fresh castor leaves treated with freshly prepared bacterial suspension until pupation. Observations were made daily on larval mortality.

### Dose response experiments

Dose response experiments were conducted with bacterial isolates, *K. pneumoniae* and *P. paralactis* based on their higher larval mortality in *S. litura* according to screening test. Five different bacterial concentrations of each bacterial isolate were used i.e. C1= 3.2 × 10^8^ cfu/ml, C2=8.2 × 10^8^ cfu/ml, C3=1.9 × 10^9^ cfu/ml, C4=3.6 × 10^9^ cfu/ml and C5=5.8 × 10^9^ cfu/ml in case of *K. pneumoniae* and C1=2.4 × 10^8^ cfu/ml, C2=6.8 × 10^8^ cfu/ml, C3=1.4 × 10^9^ cfu/ml, C4=3.2 × 10^9^ cfu/ml and C5=5.0 × 10^9^ cfu/ml in case of *P. paralactis* (based on their OD_600_ values). Ten ml of each concentration was used to treat the castor leaves (approximately 10 cm^2^). The leaves dipped in PBS only served as control. Experiments on both the bacterial cultures were conducted on 50 s instar larvae (6 days old) with 5 replications (10 larvae per replicate) for each concentration. After every 48 h diet was changed till pupation. The observations on larval mortality and development of *S. litura* were recorded daily. The percentage of adult emergence was calculated and the freshly emerged adults from all the treatments and control were transferred to oviposition jar in 2:1 ratio (2 female: 1 male) to observe the longevity and fecundity of adults. One oviposition jar represented one replicate and all the treatments were replicated thrice. Based on larval mortality data, lethal concentration (LC_50_) values for both the bacteria were determined by Probit analysis using the SPSS 20.0 statistical software.

### Nutritional analysis

To evaluate the effect of bacterial cultures, *K. pneumoniae* and *P. paralactis* on nutritional physiology, second instar larvae of *S. litura* were starved for 3–4 h. The larvae were weighed individually and released in rearing vials containing the weighed leaves treated with above mentioned bacterial concentrations. Similarly leaves dipped in PBS served as control. The experiment was performed on 50 s instar larvae in each concentration of both the bacterial cultures. After 72 h of feeding, the weight of larvae, residual diet and faecal matter was recorded and overall change in each variable was compared with the last recorded value. The data obtained were used to calculate nutritional indices on dry weight basis following the procedure of Farrar et al. [78] and Datta et al. [79]. Relative growth (RGR) and consumption rates (RCR) were calculated as *G*/*I* (*G* = change in larval dry weight/day and *I* = initial larval dry weight) and *C*/*I* (*C* = change in diet dry weight/day and *I* = initial larval dry weight) respectively. Both are calculated as mg mg^−1^ d^−1^. Index of food conversion efficiency (ECI) was calculated as 100 × *G*/*C*; where *G* = dry weight gain of insect and *C* = dry weight of food consumed. Approximate digestibility (AD) and efficiency of conversion of digested food (ECD) were calculated as *C* − *F*/*C* × 100 (where *C* = change in diet dry weight/day and *F* = dry weight of frass/day) and *G*/*C* − *F* × 100 (where *G* = change in larval dry weight/day, *C* = change in diet dry weight/day and *F* = dry weight of frass/day, respectively. Efficiency of conversion of ingested food (ECI), approximate digestibility (AD) and efficiency of conversion of digested food (ECD) were calculated as percent.

### Effect of *K. pneumoniae* and *P. paralactis* on gut microflora of *S. litura*

To determine the effect of ingestion of bacteria on gut microbial diversity of *S. litura,* second instar larvae were fed on LC_50_ values of *K. pneumoniae* and *P. paralactis.* After 96 h of bacterial treatment, ten healthy control larvae and ten infected larvae showing the symptoms of slow growth, reduction in size, black pigmentation on integument were selected. The gut of both infected and control larvae were removed separately with the help of dissection scissors. These larval guts were homogenized in a homogenizer containing 1 ml of 0.1 M phosphate buffer (pH 7.0) under the laminar flow cabinet. The homogenized suspension was diluted up to ten times and 100 µl of each dilution was spread on Luria Bertani (LB) agar plates with the help of spreader. The plates were incubated for 48 h at 30˚C and observed for appearance of bacterial colonies and the cfu/ml of different bacteria was calculated by plate count method.

### Histological analysis

The effect of LC_50_ values of *K. pneumoniae* and *P. paralactis* infection on histology of midgut of *S. litura* was studied on 2nd instar (6 days old) larvae. The leaves treated with bacterial suspensions were fed to larvae for 96 h. The larvae fed on leaves dipped in PBS only served as control. The temperature and humidity conditions were maintained at 25± 2ºC and 60 ± 5% respectively. After 96 h, larvae were dissected aseptically and gut was removed in distilled water. The gut was preserved in 10% formalin until processing of tissue. After fixation, the material was washed with distilled water in a tube and process was repeated many times. Then dehydration of tissue was done by passing through 30-90% grades of alcohol. For each treatment as well as control, the tissue was fixed in paraffin wax. After solidification of wax blocks, thin ribbons from blocks were prepared using the microtome. These thin ribbons having gut sections were placed on slide coated with very thin layer of Mayer’s egg albumin and kept on warm hot plate at 40-45ºC temperature for equal spreading of wax. Again tissue section placed on slide was passed through 30-90% grades of alcohol in ascending and descending way. Then permanent staining of slides was done by using hematoxylin and eosin stain following the methodology of Verma and Srivastava [80]. The slides were observed under the microscope (Evos XL Core) at magnification 400X to study the histology of gut tissue.

### Evaluation of the presence of bacteria in larval hemolymph

To evaluate the presence of bacteria in hemolymph of larvae, the second instar larvae were fed on LC_50_ values of *K. pneumoniae* and *P. paralactis*. After 96 h of bacterial treatment, 100 µl of hemolymph was collected from ten infected larvae of bacteria treated groups and ten control larvae. The hemolymph collected was serially diluted and spread on LB agar plates with the help of spreader. Plates were incubated at 30˚C and observed after 48 h for the appearance of bacterial colonies.

### Statistical analysis

The larval mortality, development period, adult emergence, adult deformities and all parameters of nutritional analysis were replicated five times (10 larvae/replication) while the experiments on male and female longevities, fecundity and egg hatching were replicated three times. All the values were represented as their mean ± SE. The difference in means were compared by one way analysis of variance (ANOVA) with Tukey’s test at *p* ⩾ 0.05. SPSS 20.0 software was used for statistical analysis.

## Data Availability

Not applicable.

## References

[1] Ahmad M, Arif MI, Ahmad M (2007). Occurrence of insecticide resistance in field populations of *Spodoptera litura* (Lepidoptera: Noctuidae) in Pakistan. Crop Prot.

[2] Ahmad M (2009). Observed potentiation between pyrethroid and organophosphorus insecticides for the management of *Spodoptera litura* (Lepidoptera: Noctuidae). Crop Prot.

[3] Dhaliwal GS, Jindal V, Dhawan AK (2010). Insect pest problems and crop losses: changing trends. Indian J Ecol.

[4] Punithavalli M, Sharma AN, Rajkumar MB (2014). Seasonality of the common cutworm *Spodoptera litura* in a soybean ecosystem. Phytoparasitica.

[5] Fu X, Zhao X, Xie B, Ali A, Wu K (2015). Seasonal pattern of *Spodoptera litura* (Lepidoptera: Noctuidae) migration across the Bohai Strait in northern China. J Econ Entomol.

[6] Ahmad M, Sayyed AH, Saleem MA, Ahmad M (2008). Evidence for field evolved resistance to newer insecticides in *Spodoptera litura* (Lepidoptera: Noctuidae) from Pakistan. Crop Prot.

[7] Saleem M, Hussain D, Ghouse G, Abbas M, Fisher SW (2016). Monitoring of insecticide resistance in *Spodoptera litura* (Lepidoptera: Noctuidae) from four districts of Punjab, Pakistan to conventional and new chemistry insecticides. Crop Prot.

[8] Tong H, Su Q, Zhou X, Bai L (2013). Field resistance of *Spodoptera litura* (Lepidoptera: Noctuidae) to organophosphates, pyrethroids, carbamates and four newer chemistry insecticides in Hunan, China. J Pest Sci.

[9] Sang S, Shu B, Yi X, Liu J, Hu M, Zhong G (2016). Cross-resistance and baseline susceptibility of *Spodoptera litura* (Fabricius)(Lepidoptera: Noctuidae) to cyantraniliprole in the south of China. Pest Manag Sci.

[10] Wang X, Huang Q, Hao Q, Ran S, Wu Y, Cui P, Yang J, Jiang C, Yang Q (2018). Insecticide resistance and enhanced cytochrome P450 monooxygenase activity in field populations of *Spodoptera litura* from Sichuan, China. Crop Prot.

[11] Valicente FH (2019). Entomopathogenic Viruses. natural enemies of insect pests in neotropical agroecosystems.

[12] Fernández-Grandon GM, Harte SJ, Ewany J, Bray D, Stevenson PC (2020). Additive effect of botanical insecticide and entomopathogenic fungi on pest mortality and the behavioral response of its natural enemy. Plants.

[13] Stahly DP, Andrews RE, Yousten AA (2006). The genus *Bacillus*-insect pathogens. Prokaryotes.

[14] Charles JF, Silva-Filha MH, Nielsen-LeRoux C (2000). Mode of action of *Bacillus sphaericus* on mosquito larvae: incidence on resistance. Entomopathogenic bacteria: from laboratory to field application.

[15] Mona HA, Aly NA (2009). Insecticidal activity and genetic characterization of four bacterial isolates of *Xenorhabdus* and *Photorhabdus* associated with entomopathogenic nematodes. Pest Technol.

[16] Pineda-Castellanos ML, Rodríguez-Segura Z, Villalobos FJ, Hernández L, Lina L, Nuñez-Valdez ME (2015). Pathogenicity of isolates of *Serratia marcescens* towards larvae of the scarab *Phyllophaga blanchardi* (Coleoptera). Pathogens.

[17] Ruiu L (2015). Insect pathogenic bacteria in integrated pest management. Insects.

[18] Raymann K, Coon KL, Shaffer Z, Salisbury S, Moran NA (2018). Pathogenicity of *Serratia marcescens* strains in honey bees. mBio.

[19] Tabashnik BE, Cushing NL, Finson N, Johnson MW (1990). Field development of resistance to *Bacillus thuringiensis* in diamondback moth (Lepidoptera: Plutellidae). J Econ Entomol.

[20] Zago HB, Siqueira HA, Pereira EJ, Picanço MC, Barros R (2014). Resistance and behavioural response of *Plutella xylostella* (Lepidoptera: Plutellidae) populations to *Bacillus thuringiensis* formulations. Pest Manag Sci.

[21] Naik VC, Kumbhare S, Kranthi S, Satija U, Kranthi KR (2018). Field-evolved resistance of pink bollworm, *Pectinophora gossypiella* (Saunders)(Lepidoptera: Gelechiidae), to transgenic *Bacillus thuringiensis* (Bt) cotton expressing crystal 1Ac (Cry1Ac) and Cry2Ab in India. Pest Manag Sci.

[22] Yang F, Williams J, Porter P, Huang F, Kerns DL (2019). F2 screen for resistance to *Bacillus thuringiensis* Vip3Aa51 protein in field populations of *Spodoptera frugiperda* (Lepidoptera: Noctuidae) from Texas, USA. Crop Prot.

[23] Yang F, Head GP, Price PA, Santiago González JC, Kerns DL (2020). Inheritance of *Bacillus thuringiensis* Cry2Ab2 protein resistance in *Helicoverpa zea* (Lepidoptera: Noctuidae). Pest Manag Sci.

[24] Eski A, Demir I, Güllü M, Demirbağ Z (2018). Biodiversity and pathogenicity of bacteria associated with the gut microbiota of beet armyworm, *Spodoptera exigua* Hübner (Lepidoptera: Noctuidae). Microb. Pathog.

[25] Noman MS, Liu L, Bai Z, Li Z (2020). Tephritidae bacterial symbionts: potentials for pest management. Bull Entomol Res.

[26] Engel P, Moran NA (2013). The gut microbiota of insects–diversity in structure and function.. Rev.

[27] Ceja-Navarro JA, Vega FE, Karaoz U, Hao Z, Jenkins S, Lim HC, Kosina P, Infante F, Northen TR, Brodie EL (2015). Gut microbiota mediate caffeine detoxification in the primary insect pest of coffee. Nat Commun..

[28] Warnecke F, Luginbühl P, Ivanova N, Ghassemian M, Richardson TH, Stege JT, Cayouette M, McHardy AC, Djordjevic G, Aboushadi N, Sorek R (2007). Metagenomic and functional analysis of hindgut microbiota of a wood-feeding higher termite. Nature.

[29] Douglas AE, Francois CL, Minto LB (2006). Facultative ‘secondary’bacterial symbionts and the nutrition of the pea aphid, *Acyrthosiphon pisum*. Physiol Entomol.

[30] Haloi K, Kalita MK, Nath R, Devi D (2016). Characterization and pathogenicity assessment of gut-associated microbes of muga silkworm *Antheraea assamensis* Helfer (Lepidoptera: Saturniidae). J Invertebr Pathol..

[31] Ketola T, Mikonranta L, Laakso J, Mappes J (2016). Different food sources elicit fast changes to bacterial virulence. Biol Lett.

[32] Thakur A, Dhammi P, Saini HS, Kaur S (2015). Pathogenicity of bacteria isolated from gut of *Spodoptera litura* (Lepidoptera: Noctuidae) and fitness costs of insect associated with consumption of bacteria. J Invertebr Pathol.

[33] Cakici FO, Sevim A, Demirbag Z, Demir I (2014). Investigating internal bacteria of *Spodoptera littoralis* (Boisd.)(Lepidoptera: Noctuidae) larvae and some *Bacillus* strains as biocontrol agents. Turk J Agric For.

[34] Sevim A, Demirbag Z, Demir I (2010). A new study on the bacteria of *Agrotis segetum* Schiff.(Lepidoptera: Noctuidae) and their insecticidal activities. Turk J Agric For.

[35] Perchat S, Buisson C, Chaufaux J, Sanchis V, Lereclus D, Gohar M (2005). *Bacillus cereus* produces several nonproteinaceous insecticidal exotoxins. J Invertebr Pathol.

[36] Ruffner B, Péchy-Tarr M, Ryffel F, Hoegger P, Obrist C, Rindlisbacher A, Keel C, Maurhofer M (2013). Oral insecticidal activity of plant‐associated pseudomonads. Environ Microbiol..

[37] Ramya SL, Venkatesan T, Srinivasa Murthy K, Jalali SK, Verghese A (2016). Detection of carboxylesterase and esterase activity in culturable gut bacterial flora isolated from diamondback moth, *Plutella xylostella* (Linnaeus), from India and its possible role in indoxacarb degradation. Braz. J. Microbiol..

[38] Behar A, Yuval B, Jurkevitch E. Gut bacterial communities in the Mediterranean fruit fly (*Ceratitis capitata*) and their impact on host longevity. <background-color:#CCFF99;bvertical-align:super;>J</background-color:#CCFF99;bvertical-align:super;>. Insect Physiol. 2008;54(9):1377–83. 10.1016/j.jinsphys.2008.07.011 18706909

[39] Chen B, Teh BS, Sun C, Hu S, Lu X, Boland W, Shao Y (2016). Biodiversity and activity of the gut microbiota across the life history of the insect herbivore *Spodoptera littoralis*. Sci. Rep.

[40] He Y, Qin Q, DiLegge MJ, Vivanco JM (2019). Isolation of *Klebsiella pneumoniae* and *Pseudomonas aeruginosa* from entomopathogenic nematode-insect host relationship to examine bacterial pathogenicity on *Trichoplusia ni*. Microb Pathog.

[41] Mohanta MK, Saha AK, Saleh DK, Islam MS, Mannan KS, Fakruddin M (2015). Characterization of *Klebsiella granulomatis* pathogenic to silkworm, *Bombyx mori* L. 3 Biotech.

[42] Insua JL, Llobet E, Moranta D, Pérez-Gutiérrez C, Tomás A, Garmendia J, Bengoechea JA (2013). Modeling *Klebsiella pneumoniae* pathogenesis by infection of the wax moth *Galleria mellonella*. Infect Immun.

[43] Olcott MH, Henkels MD, Rosen KL, L. Walker F, Sneh B, Loper JE, Taylor BJ. Lethality and developmental delay in *Drosophila melanogaster* larvae after ingestion of selected *Pseudomonas fluorescens* strains. PloS One. 2010;5(9):e12504. 10.1371/journal.pone.0012504 PMC2938339 20856932

[44] Chen WJ, Hsieh FC, Hsu FC, Tasy YF, Liu JR, Shih MC (2014). Characterization of an insecticidal toxin and pathogenicity of *Pseudomonas taiwanensis* against insects. PLoS pathogens.

[45] Maciel-Vergara G, Jensen AB, Eilenberg J (2018). Cannibalism as a possible entry route for opportunistic pathogenic bacteria to insect hosts, exemplified by *Pseudomonas aeruginosa*, a pathogen of the giant mealworm *Zophobas morio*. Insects.

[46] Péchy-Tarr M, Bruck DJ, Maurhofer M, Fischer E, Vogne C, Henkels MD, Donahue KM, Grunder J, Loper JE, Keel C (2008). Molecular analysis of a novel gene cluster encoding an insect toxin in plant‐associated strains of *Pseudomonas fluorescens*. Environ. Microbiol.

[47] Lam K, Babor D, Duthie B, Babor EM, Moore M, Gries G (2007). Proliferating bacterial symbionts on house fly eggs affect oviposition behaviour of adult flies. Anim. Behav.

[48] Ben Ami E, Yuval B, Jurkevitch E (2010). Manipulation of the microbiota of mass-reared Mediterranean fruit flies *Ceratitis capitata* (Diptera: Tephritidae) improves sterile male sexual performance. The ISME J.

[49] Niyazi N, Lauzon CR, Shelly TE (2004). Effect of probiotic adult diets on fitness components of sterile male Mediterranean fruit flies (Diptera: Tephritidae) under laboratory and field cage conditions. J. Econ. Entomol.

[50] Yuval B, Ben-Ami E, Behar A, Ben‐Yosef M, Jurkevitch E (2013). The Mediterranean fruit fly and its bacteria–potential for improving sterile insect technique operations. Appl Entomol.

[51] Papanikolaou G, Pantopoulos K (2005). Iron metabolism and toxicity. Toxicol. Appl. Pharmacol..

[52] Sonawane MS, Chaudhary RD, Shouche YS, Sayyed RZ (2018). Insect gut bacteria: a novel source for siderophore production. Proceedings of the National Academy of Sciences, India Section B: Biol. Sci.

[53] Xia X, Lan B, Tao X, Lin J, You M (2020). Characterization of *Spodoptera litura* gut bacteria and their role in feeding and growth of the host. Front Microbiol..

[54] Song F, Lin Y, Chen C, Shao E, Guan X, Huang Z (2016). Insecticidal activity and histopathological effects of Vip3Aa protein from *Bacillus thuringiensis* on *Spodoptera litura*. J. Microbiol. Biotechnol..

[55] Deepak T, Shashank AS, Shivaraj Y, Asiya Nuzhat FB (2019). Toxic effect of *Bacillus thuringiensis* (Serotype 14) bacteria shows behavioural & histological changes on mosquito larvae. J Entomol Zool Stud.

[56] Mason KL, Stepien TA, Blum JE, Holt JF, Labbe NH, Rush JS, Raffa KF, Handelsman J. From commensal to pathogen: translocation of *Enterococcus faecalis* from the midgut to the hemocoel of Manduca sexta. mBio. 2011;2(3):e00065–11. 10.1128/mBio.00065-11 PMC3101781 21586646

[57] Jurat-Fuentes JL, Jackson TA. Bacterial Entomopathogens. In: Vega FE, Kaya HK, editors. Insect Pathology. Elsevier; 2012. p. 265–349. 10.1016/B978-0-12-384984-7.00008-7.

[58] Andrejko M, Zdybicka-Barabas A, Cytryńska M (2014). Diverse effects of *Galleria mellonella* infection with entomopathogenic and clinical strains of *Pseudomonas aeruginosa*. J Invertebr Pathol.

[59] Sikorowski PP, Lawrence AM (1994). Microbial contamination and insect rearing. Am Entomol.

[60] Alverdy J, Holbrook C, Rocha F, Seiden L, Licheng R (2000). Gut-derived sepsis occurs when the right pathogen with the right virulence genes meets the right host: evidence for in vivo virulence expression in *Pseudomonas aeruginosa*. Ann Surg.

[61] Broderick NA, Robinson CJ, McMahon MD, Holt J, Handelsman J, Raffa KF (2009). Contributions of gut bacteria to *Bacillus thuringiensis*-induced mortality vary across a range of Lepidoptera. BMC Biol.

[62] Azambuja P, Garcia ES, Ratcliffe NA (2005). Gut microbiota and parasite transmission by insect vectors. Trends Parasitol.

[63] Jing TZ, Qi FH, Wang ZY (2020). Most dominant roles of insect gut bacteria: digestion, detoxification, or essential nutrient provision?. Microbiome.

[64] Bidari F, Shams-Bakhsh M, Mehrabadi M. Isolation and characterization of a *Serratia marcescens* with insecticidal activity from *Polyphylla olivieri* (Col.: Scarabaeidae). J Appl Entomol. 2018;142(1–2):162–72.

[65] Ryu JH, Kim SH, Lee HY, Bai JY, Nam YD, Bae JW, Lee DG, Shin SC, Ha EM, Lee WJ (2008). Innate immune homeostasis by the homeobox gene caudal and commensal-gut mutualism in *Drosophila*. Science.

[66] Robinson CJ, Schloss P, Ramos Y, Raffa K, Handelsman J (2010). Robustness of the bacterial community in the cabbage white butterfly larval midgut. Microb. Ecol..

[67] Vacheron J, Péchy-Tarr M, Brochet S, Heiman CM, Stojiljkovic M, Maurhofer M, Keel C (2019). T6SS contributes to gut microbiome invasion and killing of an herbivorous pest insect by plant-beneficial *Pseudomonas protegens*. The ISME J.

[68] Khaeso K, Andongma AA, Akami M, Souliyanonh B, Zhu J, Krutmuang P, Niu CY (2018). Assessing the effects of gut bacteria manipulation on the development of the oriental fruit fly, *Bactrocera dorsalis* (Diptera; Tephritidae). Symbiosis.

[69] Nathan SS, Chung PG, Murugan K (2005). Effect of biopesticides applied separately or together on nutritional indices of the rice leaffolder *Cnaphalocrocis medinalis*. Phytoparasitica.

[70] Buchon N, Broderick NA, Poidevin M, Pradervand S, Lemaitre B (2009). *Drosophila* intestinal response to bacterial infection: activation of host defense and stem cell proliferation. Cell Host Microbe.

[71] Chen L, Jiang H, Cheng Q, Chen J, Wu G, Kumar A, Sun M, Liu Z (2015). Enhanced nematicidal potential of the chitinase pachi from *Pseudomonas aeruginosa* in association with Cry21Aa. Sci Rep.

[72] Zhong W, Ding S, Guo H. The chitinase C gene PsChiC from *Pseudomonas sp*. and its synergistic effects on larvicidal activity. Genet. Mol. Biol<background-color:#CCFF99;bvertical-align:super;>.</background-color:#CCFF99;bvertical-align:super;> 2015;38(3):366–72. 10.1590/S1415-475738320140320 PMC4612601 26500441

[73] Loper JE, Henkels MD, Rangel LI, Olcott MH, Walker FL, Bond KL, Kidarsa TA, Hesse CN, Sneh B, Stockwell VO, Taylor BJ (2016). Rhizoxin analogs, orfamide A and chitinase production contribute to the toxicity of *Pseudomonas protegens* strain Pf-5 to *Drosophila melanogaster*. Environ Microbiol..

[74] Rao R, Fiandra L, Giordana B, de Eguileor M, Congiu T, Burlini N, Arciello S, Corrado G, Pennacchio F (2004). AcMNPV ChiA protein disrupts the peritrophic membrane and alters midgut physiology of *Bombyx mori* larvae. Insect Biochem Mol Biol.

[75] Kabir KE, Sugimoto H, Tado H, Endo K, Yamanaka A, Tanaka S, Koga D (2006). Effect of *Bombyx mori* chitinase against Japanese pine sawyer (*Monochamus alternatus*) adults as a biopesticide. Biosci Biotechnol Biochem.

[76] Suganthi M, Senthilkumar P, Arvinth S, Chandrashekara KN (2017). Chitinase from *Pseudomonas fluorescens* and its insecticidal activity against *Helopeltis theivora*. J Gen Appl Microbiol..

[77] Chandrasekaran R, Revathi K, Nisha S, Kirubakaran SA, Sathish-Narayanan S, Senthil-Nathan S (2012). Physiological effect of chitinase purified from *Bacillus subtilis* against the tobacco cutworm *Spodoptera litura* Fab. Pestic Biochem Physiol.

[78] Farrar RR, Barbour JD, Kennedy GG (1989). Quantifying food consumption and growth in insects. Ann Entomol Soc Am.

[79] Datta R, Kaur A, Saraf I, Singh IP, Kaur S (2019). Effect of crude extracts and purified compounds of *Alpinia galanga* on nutritional physiology of a polyphagous lepidopteran pest, *Spodoptera litura* (Fabricius). Ecotoxicol Environ Saf..

[80] Verma PS, Srivastava PC (2012). Advanced Practical Zoology.

